# The Relevance of the Accurate Annotation of Micro and Long Non-Coding RNA Interactions for the Development of Therapies

**DOI:** 10.3390/genes16030262

**Published:** 2025-02-24

**Authors:** Simona Panni

**Affiliations:** Dipartimento di Biologia Ecologia Scienze della Terra (DiBEST), Università della Calabria, Via Pietro Bucci Cubo 6C, 87036 Rende, Italy; simona.panni@unical.it

**Keywords:** microRNA, lncRNA, miRNA interaction network, mimics, interaction database

## Abstract

A large fraction of the human genome is transcribed in RNA molecules that do not encode for proteins but that do have a crucial role in regulating almost every level of gene expression and, thus, define the specific phenotype of each cell. These non-coding RNAs include well-characterized microRNAs and thousands of less-defined longer transcripts, named long non-coding RNAs. Both types markedly affect the onset and the progression of numerous pathologies, ranging from cancer to vascular and neuro-degenerative diseases. In recent years, a substantial effort has been made to design drugs targeting ncRNAs, and promising advancements have been produced from micro-RNA mimics and inhibitors. Each ncRNA controls several targets, and the overall effect of its inhibition or overexpression depends on the function of the set of genes it regulates. Therefore, in selecting the most appropriate target, and predicting the final outcome of ncRNA-based therapies, it is crucial to have and utilize detailed and accurate knowledge of their functional interactions. In this review, I recapitulate the principal resources which collect information on microRNA and lncRNA networks, focusing on the non-homogeneity of the data that result from disparate approaches. I highlight the role of RNA identifiers and interaction evidence standardization in helping the user to filter and integrate data derived from different databases in a reliable functional web of regulative relations.

## 1. Introduction

A key advancement in our understanding of gene regulation was the introduction of global transcriptome analyses, which revealed that most of a mammal’s genome is transcribed in RNA molecules with little or no protein coding potential [1]. The paucity of knowledge led to a rough classification of the two main classes of ncRNAs based on a general feature of RNAs, namely their lengths: short non-coding RNAs (including microRNAs, piRNAs, siRNAs, and snoRNAs) and long non-coding RNAs (including lincRNAs and eRNAs) [2,3,4].

Since then, extensive research spanning over three decades has produced a multitude of studies revealing the unexpectedly crucial role of ncRNAs in nearly all the essential biological processes [5].

In particular, microRNAs are tiny molecules measuring approximately 22 nucleotides in length that have a significant impact on gene regulation through the translational repression or degradation of target mRNAs [6,7,8]. The resulting scanty reduction of the protein product, often to less than half of the original, has a surprising effect on the proper cellular functioning, with the result that microRNA deregulation is implicated in several pathologies [9]. Countless studies have found alterations in microRNA expression levels in almost all types of cancer, through the amplification or deletion of their genes, or through epigenetic mechanisms, as well as their implication in deregulated cell proliferation, angiogenesis, and metastasis formation [10]. Some microRNAs have been classified as oncogenes (oncomiR) if their overexpression in cancer cells promotes tumor growth, or as tumor suppressor if they prevent it [11]. Genomic deletions or mutations that affect microRNA genes have been associated not only with cancer but also with several genetic disorders [12,13,14].

More complicated to catalogue, but certainly not less relevant, lncRNAs exert their influence on gene regulation in a multiplicity of ways; for example, they can interact with proteins, mRNA transcripts, or DNA itself and can act in cis or in trans on the locus they are transcribed from [15]. The precise mechanism of action has been elucidated for a small number of lncRNAs and shows that they can recruit histone-modifying enzymes to shape chromatin [16]. Interestingly, their expression is restricted to specific cell types or phases and can be altered in cancer phenotypes and other pathologies [17].

Precision medicine approaches aim to restore tampered-with molecular mechanisms, and currently, several RNA-based therapies targeting deregulated microRNAs, as well as protein-coding genes, are undergoing testing [18]. These include, among the others, small interfering RNAs (siRNAs), antisense oligonucleotides (ASOs), and short hairpin RNAs (shRNAs), which are designed to target specific mRNA molecules; some of these have been approved by the FDA and by the European Medicines Agency [19,20]. A few microRNA mimics and ASO inhibitors targeting microRNAs (antimiR) have also reached the clinical stage, although progress is slower for these products and some of the studies have been abandoned [21,22] One of the biggest challenges in this area is identifying the best microRNA candidates and predicting their secondary effects; therefore, to this end, a detailed and complete view of the interaction network of the targeted molecules is required [23]. 

In the present review, I will recapitulate the clinical use of microRNA and lncRNA, summarize the principal repositories wherein data on ncRNA interactions are conserved and made available to the users, and discuss the consequences of differences in data quality. Many tools and databases have been developed to aid with analyses and predictions concerning microRNAs’ and lncRNAs’ sequences, structures, expression, associations with diseases and so on; these are discussed in [24,25,26,27]. Repositories rarely share common guidelines and terms for annotating ncRNA data, but the development of common controlled vocabulary terms, i.e., specific words and phrases for identifying features of interest [28], greatly enhances the reuse and the integration of data, and helps researchers to select options that are biologically and clinically significant for a certain purpose. Notably, RNAcentral provides a unique access point for more than 40 repositories, as well as unique identifiers to ncRNA molecules aiding in simplifying data integration [29].

## 2. microRNAs and lncRNAs in Clinics: Biomarkers and Drug Targets

The identification of molecular markers that can help with early diagnosis, risk stratification, and the evaluation of disease stage is one of the main objectives of biomedical research, and ncRNAs have been suggested and extensively studied as potential biomarkers for the diagnosis and prognosis of numerous pathologies [30,31,32,33,34]. Based on their stability in various bodily fluids, even after being subjected to severe manipulations, and also based on the fact that their expression patterns are specific to certain diseases, microRNAs are particularly suitable for this aim. Currently, several hundreds of studies concerning the potential use of microRNAs as biomarkers are registered on the official U.S. clinical trials website (https://clinicaltrials.gov, accessed on 5 November 2024), although most of these are “observational” studies. Among them, miR-371a-3p [35,36] is under evaluation for germ cell malignancy (NCT04435756 and NCT04914026), and miR-15a and miR21 [37,38] are under investigation for colorectal cancer (NCT06738225). To increase the specificity and sensitivity of these assays, combinations of two or more microRNAs have been proposed. For example, a panel of microRNAs has been proposed to evaluate if chemotherapy should be administered or not in patients with stage II colon cancer (NT02466113), or in order to define types of thyroid cancer (NCT04285476), and miRNA expression patterns have been investigated with regard to their use in diagnosing amyotrophic lateral sclerosis (NCT01992029) [10]. In a similar manner, some lncRNAs are under evaluation, with regard to validating their potential prognostic and diagnostic value for cardiovascular, gastrointestinal, and oncological pathologies, among others [39]. For example, BACE1 is under investigation as a potential biomarker in patients with acute coronary syndrome (NCT06213493), MFI2AS1 is being investigated for patients with clear-cell kidney cancer (NCT04946266), and MALAT1 is being investigated for its potential use for oral squamous cell carcinoma (NCT05708209).

Despite some challenges in the application of micro and long non-coding RNAs as biomarkers, including their overall low levels of sensitivity, due to differences in their expression levels between different cohorts, and the lack of normal threshold levels, these molecules are promising prognostic indicators and predictive markers for treatment response, even when their molecular functions in different pathologies have not been completely assessed [40,41,42].

On the other hand, RNA-based clinical applications are not limited to their use for diagnosis, but the therapeutic targeting of ncRNAs represents a promising approach for the development of new drugs. From this perspective, the most promising advances have come from microRNA studies. Downregulated microRNAs can be replaced by mimics, which are synthetic molecules containing the sequence of their endogenous counterpart that can be delivered into cells to restore the function of tumor suppressor microRNAs. OncomiRs or pathologically overexpressed microRNAs, instead, can be inhibited by molecules carrying a complementary sequence, such as antisense oligonucleotides (ASOs) or AntagomiR, which contain a 2′-O-methoxyethyl modification [20,43]. The first attempt to administer a mimic, which was derived from the tumor suppressor miR34a and named MRX34, was ruinous, as it induced serious adverse immunological reactions, but it did demonstrate the dose-dependent regulation of oncogenic target genes in human white blood cells [44]. MicroRNA16 was identified as a tumor suppressor in chronic lymphocytic leukemia (CLL) [45], and it was subsequently associated with other types of cancer, such as lung, pleura, breast, and gastric cancer [10]. A mimic derived from the tumor suppressor miR16, named TargomiR, was fairly well-tolerated when encapsulated in bacterially derived delivery nanocells (EDV), targeted toward EGFR [46]. TargomiR was tested on malignant pleural mesothelioma and lung cancer [47]. INT-1B3 (NCT04675996) was developed from miR193a-3p and demonstrated anti-tumor activity in mice [48]. Promising results were shown by MRG-201 (NCT02603224) and MRG-229, both of which were derived from miR29, against contrast cutaneous fibrosis and idiopathic pulmonary fibrosis, respectively [49].

Several microRNA inhibitors have also entered clinical trials. Miravirsen is an LNA-antisense oligonucleotide that targets miR-122, which is essential for HCV (hepatitis C virus) replication [50]. RG-101 N-acetylgalactosamine-conjugated oligonucleotide also antagonizes miR-122 against hepatitis virus. The Miragen Therapeutics company, recently renamed Viridian Therapeutics, has developed several microRNA-based drugs, including MRG-106, also known as Cobomarsen. Cobomarsen is recommended for use in subjects with mycosis fungoides and in patients with T-cell leukemia or B-cell lymphoma. It inhibits miR-155, an oncogenic and pro-inflammatory microRNA that is often upregulated in malignant T and B cells [51]. Viridian has also produced MRG-110, a locked nucleic acid-based inhibitor of miR-92a-3p that has been shown to have therapeutic effects on wound healing in mice [52] and to upregulate miR-92a targets such as *ITGA5* and *CD93* in human patients [53]. MGN-9103, targeting microRNA-208, is under evaluation as a treatment for chronic heart failure [54,55].

RGLS4326 is an inhibitor of miR17 that attenuates cyst growth in autosomal dominant polycystic kidney disease (ADPKD) [56]. The microRNA17 family is upregulated in ADPKD; its deletion was found to impair embryogenesis in mice embryos but did not impact lifespan in adult mice. RGLS326 was proposed in 2019 as an effective treatment for upregulating *PKD1* and *PKD2* (miR17 targets) in mouse models [56], and, in 2022, the Regulus Therapeutics announced positive and safety data from phase 1 trials. A phase 1b study was conducted in which antisense therapy targeting miR-132 (CDR132L) was administered in patients with heart failure; this was well tolerated in combination with linear plasma pharmacokinetics [57].

Other microRNA candidates are progressing in clinical trials, as extensively described in [18,58], although none have been yet approved for commercial use, and several (including Miravirsen) have been halted. The main challenges comprise delivery to specific cells and the potential for the degradation of oligonucleotides. In recent years, several strategies have been developed to enhance the RNA stability and to reduce adverse immune reactions that are caused by chemical modifications of the nucleotides and by the nanoparticle encapsulation used for delivery [20,43].

On the contrary, no lncRNA-targeting drug has yet reached advanced phases of clinical trials, although the rapid progress currently being made in research suggests that they could be very promising candidates. The inhibition of oncogenic lncRNA HOTTIP against hepatocellular carcinoma (NCT06544005) and of BACE1/BACE1-AS against heart failure (NCT06213493) is under evaluation.

In order to identify the most efficacious therapeutic target, to predict its global effect, and to ensure appropriate controls are in place during clinical trials, it is necessary to have very detailed knowledge of the direct and indirect effects of ncRNAs on the regulative network, namely knowledge of the interactions that occur in a specific cell type or organism.

**Figure 1:**
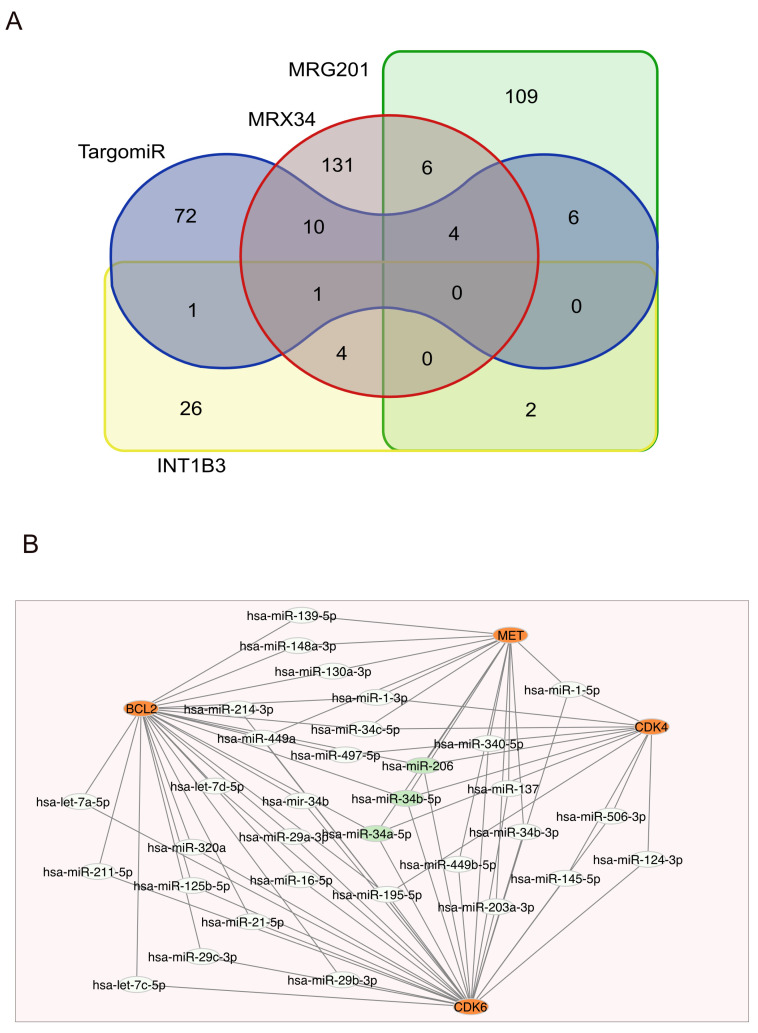
Overlapping targets in microRNA regulation. (**A**) Overlapping targets of the mimics MRX34, TargomiR, MRG-201, and INT-1B3 according to genes targeted by their natural counterparts (hsa-miR-34a-5p, hsa-miR16-5p, hsa-miR-29a-3p, and hsa-miR-193a-3p, respectively). The list of considered interactors was retrieved from four datasets filtered for “strong interaction” and ”luciferase assay”, as previously reported in [60]. The Venn diagram was drawn using https://bioinformatics.psb.ugent.be/webtools/Venn/, accessed on 10 December 2024. (**B**) MicroRNA network regulating *BCL2*, *CDC4*, *CDC6*, and *MET*, four genes suggested to cause the tumor-suppressive effects of miR34a. The network shows only the microRNAs that regulate at least two of the four genes. MiR-34a, miR-34b, and miR-206, which are highlighted in green, regulate all four genes.

It is well known that both oncomiRs and tumor suppressors target multiple genes and may have common targets [59,60]. Figure 1A shows the overlapping targets of the four mimics described above. Although most of the repressed genes differ, it is worth noting that fourteen genes are regulated by MRX34 and TargomiR, including *Notch*, and that four of those genes (*Cdk6*, *Bcl2*, *Vegf*, and *Myb*) are also regulated by MRG201, suggesting that the three mimics may have some common effects. The figure also shows a subgraph of the microRNA network regulating four MRX34 targets (Figure 1B).

To reduce off-target effects, small molecules or oligos inhibiting one specific interaction of a micro or long ncRNA with a target have been developed [20]. Among these, target site blockers specifically inhibit one microRNA–mRNA interaction without affecting other genes, but their clinical use is still limited [61].

## 3. microRNA Network: The State of the Art

Mature microRNAs are incorporated into the RNA-induced silencing complex (RISC) and guide it toward transcripts with sequences complementary to the seed [8,62]. Each microRNA can affect a broad spectrum of targets, from one target to over one hundred targets, and determines its final effect. The inhibition or enhancement of a microRNA with synthetic molecules perturbs the network of causal and physical interactions, and it may have unexpected results if the network has not been previously completely elucidated [22]. The most obvious (and time-consuming) way to gain information about a microRNA interaction web is to analyze it through small-scale experiments conducted in the appropriate cell types. Thousands of articles have made contributions to elucidating the functions of the most-studied microRNAs. To gather all this information, dedicated resources collect binary interactions from the literature to make them easily accessible to readers; these can be either integrated or not with the results from high-throughput approaches and with predictions. However, it can be a discouraging task for the user to decide among multiple options of what to consider a bona fide interactor. 

### 3.1. Common Approaches for Studying microRNA–mRNA Interactions

**Figure 2:**
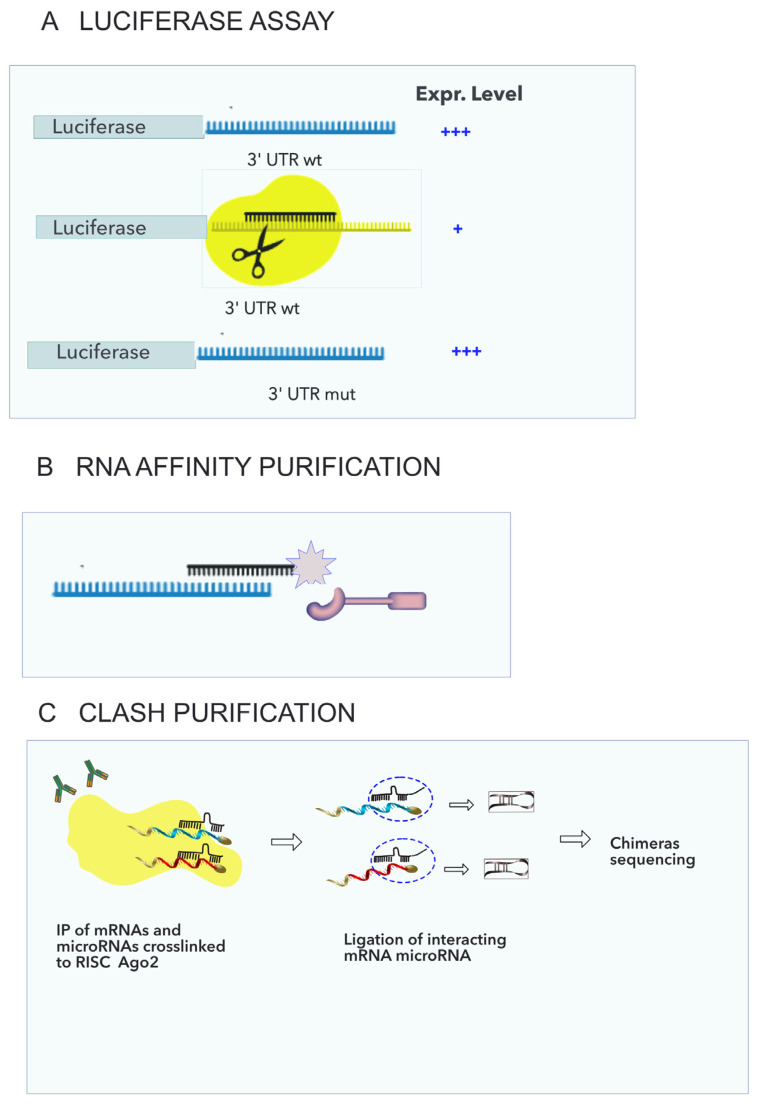
Technical approaches for detecting microRNA–mRNA interactions. (**A**) Luciferase assay. The 3′UTR of a gene is fused to the luciferase gene, and the construct is transfected in cells with or without the microRNA mimic. The luciferase expression level of the wt gene is compared with the mutated form (where the sequences complementary to the seed are deleted), and this is used to demonstrate (or to not demonstrate) the binding. (**B**) Affinity purification. RNA is linked to a tag, and the complex is purified and sequenced. (**C**) Crosslinking ligation and sequencing of hybrids (CLASH). A protein in the RISC complex is crosslinked to RNAs and immunoprecipitated. The mRNA-microRNA pairings associated with the protein are truncated, and the ends of the RNA duplexes are ligated. The ligated chimeras are then sequenced to identify RNA-RNA interactions. Some icons used in this figure have been downloaded from Reactome icon library [75].

Among the most common approaches for studying microRNA targets are luciferase assay and RNA affinity purification, which demonstrate the binding of the microRNA to the mRNA (Figure 2A,B), while measuring decrease in mRNA or protein, upon transfection of the microRNA, demonstrates a functional interaction that may involve other molecules.

In more detail, in the luciferase assay, the 3′ UTR containing the potential seed sequence of the mRNA is cloned and fused to a reporter gene, and the expressed amount is measured with or without the corresponding mimic. The reduction in luciferase expression proves that the microRNA has bound to the seed sequence if the mutation analysis confirms that it depends on the presence of the specific seed sequence [63,64,65].

A genome-wide method was developed in 2003 to identify which transcripts are bound to a certain protein using ultraviolet crosslinking followed by the immunoprecipitation of the protein [66]. The interacting protein was covalently linked to the RNAs, and the complex was then purified from cell lysates. The method, named CLIP (crosslinking immunoprecipitation), became very popular when it was combined with next-generation sequencing of the bound molecules. At least three variants have been developed: high-throughput sequencing, HITS-CLIP; photoactivatable ribonucleoside-enhanced, PAR-CLIP; and individual nucleotide resolution, iCLIP [67]. This method is commonly used to immunoprecipitate pools of microRNAs and mRNAs which are bound to one of the proteins in the RISC complex, but it does not allow us to discern specific microRNA–mRNA pairs. It is debated whether it can be used as a proof of RNA-RNA interaction when combined with a prediction tool.

A further modified version of the CLIP was developed to focus on RNA-RNA interactions with the CRAC (UV crosslinking and analysis of cDNA), wherein the chimeric RNA hybrids occasionally formed during the crosslinking were sequenced [68]. Subsequently, the efficiency of chimera recovery was improved, and the CLASH protocol was developed [69]. In principle, the linked RNAs are bound together in the physiological complex, so that the sequencing of the chimeras enables the rescue of true microRNA–mRNA interactions (Figure 2C).

The peculiar mechanism used by microRNAs to select their partner, i.e., binding them with the seed sequence located at the 5′ of the microRNA that is complementary to a short region in the transcript, makes them superbly suitable for binding prediction methods. It must be recalled that almost all microRNA studies, including both small- and large-scale experiments, start with predictions, to circumscribe the investigation or to identify the binding region. However, predictions alone largely overestimate the number of potential binding sites. Some resources have collected the lists of microRNAs and mRNAs that have been immunoprecipitated in CLIP and pair them with the help of predictions; alternatively, prediction tools can utilize CLIP data to improve their performance. Expression profiles are also taken into account to train predictors. Overall, it is debated whether this method can be considered a weak experimental validation or a sound inference. A huge number of tools return microRNA targets entirely based on these prediction algorithms, as reviewed in [70].

### 3.2. Curation of ncRNA Interactions in Public Databases

When information is extracted from the original paper and condensed into an informatic accessible format, arbitrary decisions are inevitably taken on how to interpret and store the data, both if the work is carried out manually or using text-mining algorithms. It is often unclear to the end-user what is really stored in the database, especially when data from high-throughput experiments appear beside stronger evidence.

A similar issue faced the field of protein networks more than 20 years ago, and the major providers of protein–protein interactions (PPIs) congregated as the International Molecular Exchange Consortium (IMEx) in order to develop a common file format to represent protein-interaction data, and common guidelines [40]. Nowadays, the IMEx Consortium is recognized as a Core Data Resource for both Elixir (https://elixir-europe.org/, accessed on 2 January 2025) and Global Core Biodata (https://globalbiodata.org/what-we-do/global-core-biodata-resources/ accessed on 2 January 2025) and provides non-redundant interaction data presented in full details and described using controlled vocabulary terms. Each interaction is equipped with a score of reliability, based on the kind of experiment used to demonstrate it, and the number of times it has been observed [71]. Although it is mainly focused on protein interactions, in recent years, the IntAct database [72], which is part of the IMEx Consortium, has collected ncRNA interactions, following the same criteria used for protein networks [65,73]. According to the IMEx criteria, to permit its annotation in the interaction database, the binding of a microRNA to its target must be demonstrated at the experimental level. As a consequence, not all the evidence can be annotated: experiments demonstrating the functional (i.e., causal) relationship but not the binding must be discarded.

Similar criteria were adopted in the annotation process created by the Gene Ontology Consortium for the term “mRNA binding involved in posttranscriptional gene silencing” [74]. In general, this gene ontology condenses experimental data that demonstrate the molecular function of a gene or the biological process in which it is involved, in specific terms (GO terms) developed by the GO Consortium, and an easily accessible format for researchers and for bioinformaticians to perform analyses [75]. The UCL functional annotation group has focused on the annotation of human microRNA–mRNA interactions by extending annotations using the has input extension to specify the target gene [76]. Those interactions are available in PSICQUIC View (http://www.ebi.ac.uk/Tools/webservices/psicquic/view/main.xhtml, accessed on 2 January 2025) and can be found by selecting the EBI-GOA miRNA collection.

Other resources which collect microRNA interactions adopt much less stringent rules (Table 1).

The database miRTarBase [77] is among the most used and comprehensive databases and has collected more than three hundred thousand interactions involving microRNAs, most of which come from high-throughput microarrays and next-generation sequencing; fewer than 10% of these interactions have been demonstrated in small-scale experiments, with procedures such as luciferase assay, qRTPCR, and RNA affinity purifications. The experimental evidence and the paper from which it has been extracted are clearly specified for each entry, so that the users can filter the results according to their needs. Similarly to mirRTarBase, RNAInter [78] collects both small-scale and large-scale experiments and enables the filtering of the results. Both databases integrate data concerning RNA expression, disease association, etc., from other sources.

In both miRTarBase and RNAinter, the predicted binding sites are indicated, even when no experimental findings are listed as proof of this. On the contrary, the set of interactions collected in IntAct is displayed with the results of the mutation analysis performed in the experimental work (when available), thereby proving that the interaction occurs there. 

Another resource that provides microRNA targets is RAIN [79], which combines experimental data, including both CLASH and CLIP; interaction predictions obtained using several predictors; and literature mining to automatically retrieve interactions. A score is given to each interaction, which helps the user to filter results. The peculiarity of RAIN is that it interfaces with STRING [80], integrating microRNA interactions in the protein network.

ENCORI, previously named starBase, comprises more than 2500 CLIP-seq large-scale dataset to interpret RNA–protein and RNA-RNA binding [81].

While all of these resources are certainly crucial to improving research and models on microRNA networks, the inclusion of weak, nonexistent, or text-inferred interactions may dramatically impact the choice of therapeutic candidates and the impact assessment of drug perturbation. It is worth mentioning that isoform transcripts from the same gene may have 3′UTR of different lengths in different cell types, either containing or not containing the seed sequence. Moreover, since the number of target sequences largely exceeds microRNA abundance, the repression may be sensitive to the expression level of the microRNA [62].

**Table 1:** Principal microRNA and lncRNA interaction resources. The “Summary Features” column refers to the interactions and do not consider other reported data.

Database Name	Summary Features	Web Link	Reference
miRTarBase	MicroRNA interactions verified in low scale or predicted from large scale High coverage	https://awi.cuhk.edu.cn/~miRTarBase/miRTarBase_2025/php/index.php, accessed on 1 February 2025	[77]
RNAInter	MicroRNA and lncRNA interactions verified in low scale or predicted from large scale High coverage	http://www.rnainter.org, accessed on 1 February 2025	[78]
IntAct	Manual curation with controlled vocabulary terms Low coverage	https://www.ebi.ac.uk/intact/home, accessed on 1 February 2025	[72]
QuickGO	Manual curation with controlled vocabulary terms Low coverage	https://www.ebi.ac.uk/QuickGO/annotations, accessed on 1 February 2025	[82]
RAIN	Predicted and verified microRNA and lncRNA interactions integrated in STRING PPI network	https://rth.dk/resources/rain/, accessed on 1 February 2025	[79]
ENCORI	MicroRNA and lncRNA interactions with RNA and proteins from CLIP-seq	https://rnasysu.com/encori/, accessed on 1 February 2025	[81]
NPInter	LncRNA–chromatin interactions from large scale studies High coverage	http://bigdata.ibp.ac.cn/npinter5/, accessed on 1 February 2025	[83]
LncRNAWIKI	IncRNA interactions with proteins, genes, and microRNAs annotated with controlled vocabulary terms High coverage	https://ngdc.cncb.ac.cn/lncrnawiki/, accessed on 1 February 2025	[84]
RNAcentral	Centralized database of non-coding RNA sequences collated from expert non-coding RNA databases, model organism databases, and sequence accession databases	https://rnacentral.org, accessed on 1 February 2025	[29]

## 4. MicroRNA Network Perturbation: The Uncertainty of Quicksand

Most of the microRNA-based drugs which have entered clinical trials have been halted for adverse reactions to nuclei acids or to the delivery particles, or for insufficient effects. While progress is being made to overcome major technical issues and last-generation drugs are becoming more appealing, our current comprehension of the final effect of microRNA network perturbation still remains incomplete, and, even worse, our knowledge on regulatory interactions is scattered among several disjointed sources. Let us consider, as an example, the well-known microRNA132, which is targeted by the CDR132L drug.

**Figure 3:**
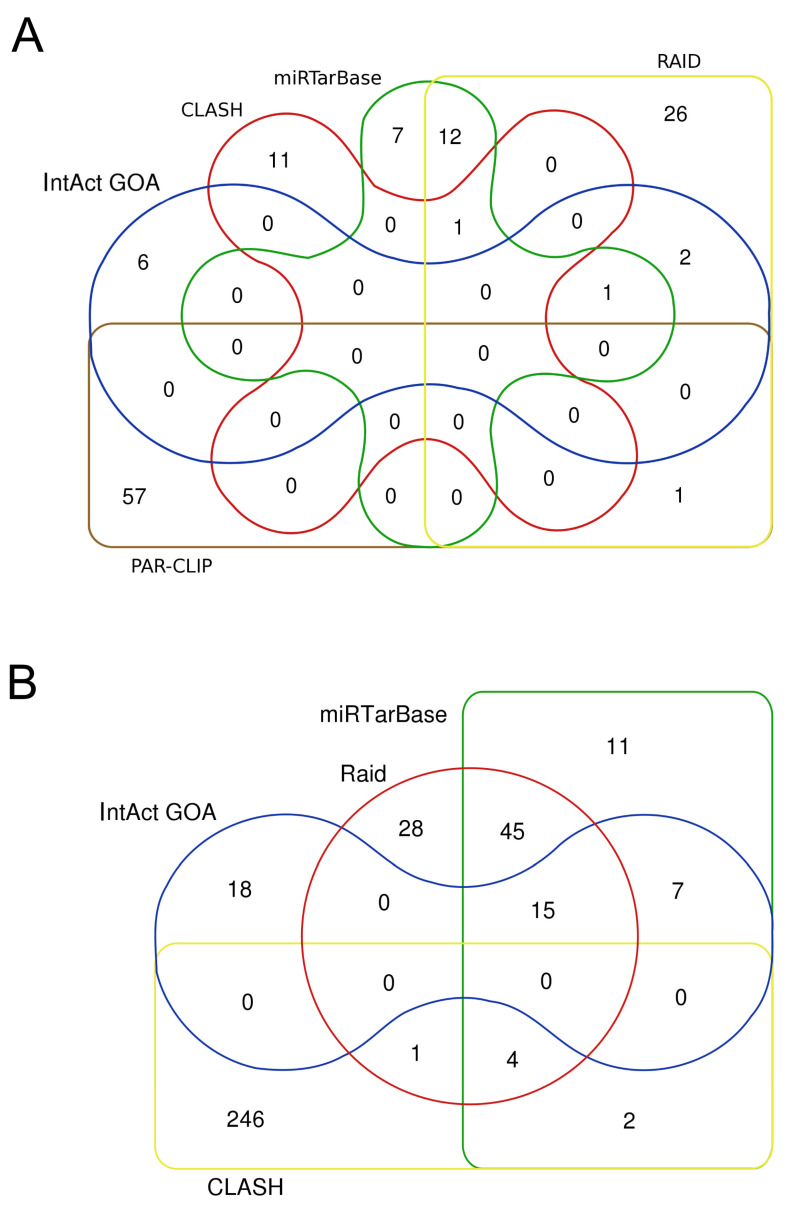
MicroRNA targets according to different datasets. (**A**) Human microRNA-132-3p targets (**B**) and microRNA-17-5p targets. The figure shows the limited overlapping among different resources or technical approaches. IntAct/EBI-GOA targets were manually curated from the literature and filtered for “luciferase assay” ([44,47]), the miRTarBase dataset was filtered for “luciferase assay” and RNAinter (Raid) for “strong evidence” [48,55], CLASH interactions were downloaded from IntAct [65], and PAR-CLIP was downloaded from miRTarBase.

According to the literature, miR132 is highly expressed in the brain, where it targets *PTEN*, *FOCO3A*, *RBFOX1*, *GSK3B*, and *Calpain2*, and it is downregulated in Alzheimer’s disease [85]. Its reintroduction has been suggested as a potential therapy for neurological disorders [86]. It is also downregulated in several cancer types [87]. On the contrary, levels of miR132 are increased in patients with heart failure, and, as described above, an inhibitory antisense is under evaluation for its potential to attenuate cardiac problems. However, considering the other described functions, its long-term effects should be monitored. To outline a complete miR-132 network, I downloaded interactions from different resources and compared them. The results are illustrated in Figure 3A and are detailed in Table 2: the overlapping genes represent a limited fraction, even when filtering for one experimental approach, and the result is even more confusing when comparing different techniques.

Other candidates for microRNA-based drugs may have a higher number of interacting genes. MicroRNA 17-5p has more than 130 interactors, identified in small-scale experiments (Figure 3B), approximately 250 were found using CLASH [88] and 600 in CLIP-seq experiments. Its inhibition of RGLS4326 to help autosomal dominant polycystic kidney disease may affect many other genes, in addition to *PKD1* and *PKD2*.

**Figure 4:**
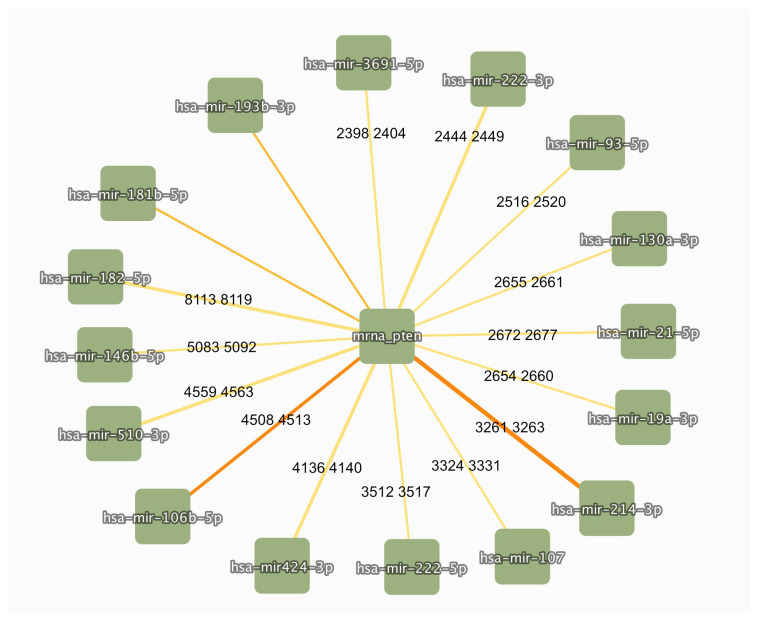
MicroRNA-*PTEN* regulatory network. MicroRNAs interacting with *PTEN* were downloaded from IntAct (accessed 20 January 2025). Binging regions inferred from mutation analyses are reported on the edges, if available (boundaries refer to ENST00000371953 transcript).

It is worth noting that key signaling proteins or transcription factors are regulated by multiple microRNAs, so that the final expression level is a result of the combined actions of different regulators, suggesting that the success of a mimic or inhibitor may rely on the context of co-expressed miRNAs. Figure 4 shows some microRNAs regulating the Phosphatase and Tensin Homolog *PTEN*, a well-known tumor suppressor gene, which inhibits the PI3K/AKT growth signaling pathway [89].

## 5. LncRNA Regulatory Network

Although there is some uncertainty concerning the microRNA interaction network, the whole set of human microRNA sequences has been identified and is catalogued in miRbase [90] and in RNAcentral [29]; therefore, there is little ambiguity as to which sequence corresponds to a microRNA name [23]. The annotation of lncRNA transcripts, instead, is more akin to a jungle, with thousands of sequences having been mainly derived from high-throughput RNA-seq data, and most of the transcripts having longer or shorter variants that are both annotated or not annotated within the reference databases [15,91]. The HUGO Gene Nomenclature Committee has worked with experts in the field to ensure a standardized nomenclature for more than 5000 human lncRNAs [92], naming genes from the RefSeq [93] and Genecode projects [94], and it is directly linked with the Ensembl, RNAcentral, and NCBI gene databases [95,96]. Databases focused on lncRNA sequences, including those that provide and those that do not provide expression data, are reviewed in [97]. It has been demonstrated that some long non-coding transcripts have the capability to regulate gene expression at multiple levels by interacting with complementary sequences on the DNA, on the RNA transcribed from the target, and with protein complexes, including chromatin remodeling enzymes [16]. Although there is no doubt as to their cooperation with transcription factors to shape the destiny of a specific cell type, the complexity of their action, possibly including the alteration of DNA fiber and the facilitating of chromatin looping, makes the experimental validation of binary functional interactions difficult. Only a handful of lncRNAs are functionally characterized with detailed knowledge on how they exert their function. Therefore, most of the repositories which collect interaction data heavily rely on predictions and high-throughput data on protein–lncRNA binding [98]. Several tools provide in silico prevision for interactions with proteins, as reviewed in [27], while tools predicting microRNA–mRNA pairings are also able to predict the interactions of microRNAs with lncRNAs. Elixir bio.tools lists 136 tools to help with investigation into lncRNAs, most of which collect expression or localization data, involvement in pathologies, and the predicted interactions or functions (https://elixir-europe.org/platforms/tools, accessed on 2 January 2025). ENCORI and RNAInter, which were mentioned in the previous paragraph for microRNAs, also provide large-scale data on proteins binding to lncRNAs, based on CLIP experiments. NPInter [83] collects large-scale, ChIRP data (chromatin isolation by RNA purification [99]) to localize genomic regions where lncRNAs are bound. RAIN utilizes information from NPinter and other evidence and, as mentioned for microRNAs, integrates it with STRING PPI data. LncRNAWIKI2.0 [84] contains information for more than 2000 functional lncRNAs gathered from other databases or annotated from the literature, using HNGC nomenclature and controlled vocabulary terms, which are completed with reference papers. To give an example, when querying the database with HOTAIR [100], one of the most studied lncRNAs, LncRNAWIKI2.0, supplies a list of 329 interactors, including proteins, microRNAs, and genes regulated through the PCG complex. It also specifies the causal effect, specifically if it is an inhibition or an enhancement. However, the accuracy may be ambiguous: for instance, according to the annotation, the reference paper [101] should contain the experimental evidence for the *KIT*, *CCND1*, *MDM2*, and *DNMT3A* genes that are regulated by HOTAIR through the PCG complex. The paper, instead, demonstrates that HOTAIR functions as a sponge to sequester microRNA 193a, which, as demonstrated elsewhere, downregulates *KIT*, *CCND1*, *MDM2*, and *DNMT3A*. This difference may either be irrelevant or dramatic depending on the user’s needs. 

Querying the other resources with the same lncRNA (human, no filters), RNAInter returns a list of 1800 proteins, DNA genes, and microRNAs that can be filtered for a reliability score; NPInter provides a list of 104 proteins, microRNAs, and lncRNAs completed with the experimental evidence; and RAIN returns a list of 10 interacting genes, predicted using text mining, which are related to the lncRNA (and in turn interact with other genes). 

As recalled above, all of these repositories provide optimal assistance to guide an investigation or to gain more insights into general network properties, but they may not be detailed enough to be used when trying to design a therapy. 

Both the IMEx consortium and the GO consortium have started to propose terms for lncRNAs annotation, but the coverage is still very limited [102]. In an ideal blue-sky scenario, lncRNAs–gene relationships should be annotated in standardized format arranged in concert with GRN (gene regulatory network) repositories, where transcription factor regulatory interactions are annotated, in order to provide a complete picture on how genomic information determines which genes are active, thus governing cellular functions [103].

## 6. Conclusions

The advent of large-scale technologies has enabled molecular biosciences to greatly improve comprehension of cell physiology, shifting the focus of the research from single molecules to complexes and networks. Moreover, they have proven that most of the human genome is expressed in non-coding RNA molecules, which ought to be integrated into networks and pathways to define regulatory models.

The final expression level of each human gene in a particular cell type or physio-pathological state is the result of an intricate network of regulation, with multiple transcription factors and lncRNAs acting on its promoter, followed by microRNA post-transcriptional regulation. Each regulator, however, is in turn regulated, sometimes in feed-forward loops, and several genes involved in transcriptional regulation are highly controlled at the post-transcriptional level [104].

An increasing number of studies on RNA interactions are being continuously published, and the data presented should be gathered and integrated with protein interactions and gene regulatory network (GRN) data. Agreements on widely accepted controlled vocabulary terms to record interaction features in unified modality, shared among different resources to facilitate data reuse, are still limited, and the cross-talk between groups working on ncRNA annotation, protein interactions, and transcription factors–genes relationships is still confined to specific projects. More connections between public biological databases would certainly help users, and a great benefit would come from the evaluation of the reliability of each interaction made by expert scientists with a strong background in the involved molecules. This policy is already in use in several resources collecting other types of data, such as data on biological pathways or gene–disease associations [105,106], and could be applied to microRNAs and lncRNAs data to filter them and to merge low- and high-throughput screenings.

Recently, more precise ontology terms to describe non-coding RNA functions have been introduced to improve ncRNA annotation [102]. To our knowledge, no attempts to annotate circular RNA interactions (or sequences) in standardized format have yet been made. Despite the evident obstacles, the success of the well-known FAIR initiative, striving for the Findability, Accessibility, Interoperability, and Reusability of data, demonstrates that the scientific community is perfectly aware that good data management is a precondition to support research and discovery [107]. Challenges outlined in this review can be addressed through a joint effort and collaboration between complementary databases.

## Figures and Tables

**Table 2:** List of hsa-miR-132-3p according to different resources.

Resource	Common Interactors	Gene Name
IntAct/EBI-GOA, RNAinter miRTarBase	1	*CDKN1A*
CLASH RNAinter miRTarBase	1	*FOXO1*
IntAct/EBI-GOA RNAinter	2	*SPRED1 RASA1*
RNAinter miRTarBase	12	*TLN2 HBEGF MAPK1 TJAP1 SOX5 ARHGAP32 RB1 HN1 SIRT1 IRAK4 CRK KLHL11*
PAR-CLIP miRTarBase	1	*OCLN*
IntAct/EBI-GOA	6	*NOS1 VCAM1 FLT1 GAB1 SPRY ESM1*
CLASH	11	*CSTF3 ANXA2 PDLIM7 IRAK1 USP22 RPSA RPL7 PARP10 RACK1 RPS5 NCKAP1*
miRTarBase	6	*GSK3B SNHG16 FOXO3 RAF1 MECP2 EGFR*
RNAinter	27	*CALU MECP2 MMGT1 NDUFB10 PAIP2 EIF2S3 TSPAN6 TMEM106B SPRY1 PXN YAP1 DPYSL3 ANO1 DPY19L1 STMN1 SDF2 DAZAP2 DCBLD2 YWHAG ESYT2 SOX4 TMEM136 AK3 MAPK3 NAP1L1 PIK3R3 BRI3*
PAR-CLIP	51	*OLFML2A HS3ST3B1 ARID2 STAG1 HOOK3 CRTC1 CD226 SLC38A2 BMPER VMP1 GID4 RAB18 TCEB1 ZNF724P KPNA1 AMD1 USP8 EML4 CCDC169 FZD6 KDM5C ALKBH4 ACSL4 CYP20A1 PFAS TRUB1 ZNF280B UBXN2A BRWD1 CHAC1 LIFR IER3IP1 GNB1 ZNF711 RAB5B TSPAN12 PHF20L1 DCAF17 ART4 Vmp1 TWISTNB PRDM15 NUP50 C6orf106 PLAGL2 SLC25A32 PARP11 CALN1 HOXC4 APBA1 PRAMEF1*

## Data Availability

All data are available in public repositories.

## Abbreviations

The following abbreviations are used in this manuscript:

FDA	Food and Drug Administration
ASO	antisense oligonucleotides
UTR	untranslated
GO	gene ontology
PPIs	protein–protein interactions
IMEx	International Molecular Exchange Consortium
qRTPCR	Quantitative Reverse Transcription Polymerase Chain Reaction
CLIP	crosslinking immunoprecipitation
HITS-CLIP	high-throughput sequencing
PAR-CLIP	photoactivatable ribonucleoside-enhanced
iCLIP	individual nucleotide resolution
ChIRP	Chromatin Isolation by RNA purification
